# Non-insulin-based insulin resistance indexes in predicting severity for coronary artery disease

**DOI:** 10.1186/s13098-022-00967-x

**Published:** 2022-12-17

**Authors:** Yu Zhang, Ruiling Wang, Xuelian Fu, Haiyan Song

**Affiliations:** Department of Endocrinology and Diabetes, The Second Affiliated Hospital of Harbin Medical University, Harbin, China

**Keywords:** Coronary artery disease, Triglyceride and glucose index, Triglyceride glucose-body mass index, Triglyceride to high-density lipoprotein cholesterol ratio, Metabolic score for insulin resistance

## Abstract

**Background:**

Triglyceride and glucose (TyG) index, triglyceride glucose-body mass (TyG-BMI) index, triglyceride to high-density lipoprotein cholesterol (TG/HDL-C) ratio, and metabolic score for insulin resistance (METS-IR) are considered simple and reliable indicators of insulin resistance (IR). Although they have been associated with coronary artery disease (CAD), evidence supporting this is limited. Here, this is the first study to demonstrate the relationship between TyG-BMI index and CAD severity. The performance of the four non-insulin-based IR indexes in predicting CAD severity was explored.

**Methods:**

We retrospectively analyzed 485 CAD patients between August 2020 and August 2021 in China, who were assigned into single- and multi-vessel CAD groups according to the coronary angiography (CAG) results. All patients were stratified into groups based on the tertiles of the TyG index, TyG-BMI index, TG/HDL-C ratio, and METS-IR.

**Results:**

Patients in the multi-vessel CAD group had significantly higher TyG index, TyG-BMI index, TG/HDL-C ratio and METS-IR than those in the single-vessel CAD group. After adjusting for confounding factors, these four indicators were significantly associated with the risk of multi-vessel CAD. Notably, the highest tertile of TyG index, TyG-BMI index, TG/HDL-C ratio and METS-IR were significantly associated with the risk of multi-vessel CAD compared to participants in the lowest tertile. We also constructed receiver operating characteristic (ROC) curve, to assess CAD severity. The area under the curve (AUC) of the ROC plots was 0.673 (95% CI 0.620–0.726; *P* < 0.001) for TyG index, while those for the TyG-BMI index, TG/HDL-C ratio, and METS-IR were 0.704 (95% CI 0.652–0.755; *P* < 0.001), 0.652 (95% CI 0.597–0.708; *P* < 0.001), and 0.726 (95% CI 0.677–0.775; *P* < 0.001), respectively.

**Conclusions:**

TyG-BMI index is not only significantly associated with CAD severity, but is also an independent risk factor for multi-vessel CAD. The TyG index, TyG-BMI index, TG/HDL-C ratio, and METS-IR could be valuable predictors of CAD severity. Among the four non-insulin-based IR indexes, METS-IR had the highest predictive value, followed by TyG-BMI index.

## Introduction

Coronary artery disease (CAD) causes a huge health and economic burden on medical systems around the world, with a high risk of death [1], a phenomenon that negatively impacts families. In recent years, the discovery and prevention of coronary heart disease (CHD) risk factors has significantly reduced prevalence and mortality of CHD, but the number of with CHD patients is still relatively high [2]. Therefore, more sensitive and easier predictors need to be sought to identify the severity of CAD in advance and to accurately formulate early intervention strategies.

Rapid development of unhealthy lifestyles and diet cultures has gradually increased the incidence of insulin resistance (IR) in recent years [3]. IR, which refers to the reduction of tissue response to insulin stimulation, has been shown to cause an imbalance in glucose metabolism, chronic hyperglycemia, oxidative stress and inflammation reaction, thereby affecting cardiovascular damage [4]. Previous studies have shown that IR is not only significantly associated with development and progression of CAD, but also with increased risk of adverse cardiovascular events [5–7]. Although previous measures of for controlling IR were based on insulin levels, the process was both complicated and costly. Consequently, researchers have adopted triglyceride and glucose (TyG) index [8], triglyceride glucose-body mass (TyG-BMI) index [9], triglyceride to high-density lipoprotein cholesterol (TG/HDL-C) ratio [10, 11], and metabolic score for insulin resistance (METS-IR) [12] as simple and reliable substitutes for IR. Previous studies have shown that TyG index, TG/HDL-C ratio and METS-IR are related to the severity of coronary lesions in CAD patient [2, 13, 14], with limited evidence. Further, TyG-BMI index was associated with increased risk of cardiovascular disease (CVD) and stroke [15–17], to date, however, nothing is known regarding the relationship between TyG-BMI index and CAD severity.

The aim of this study was to investigate the relationship between TyG-BMI index and CAD severity, and elucidate its predictive value. Furthermore, we sought to compare the value of TyG index, TyG-BMI index, TG/HDL-C ratio and METS-IR in predicting the severity of CAD.

## Methods

### Study population and selection criteria

**Fig. 1:**
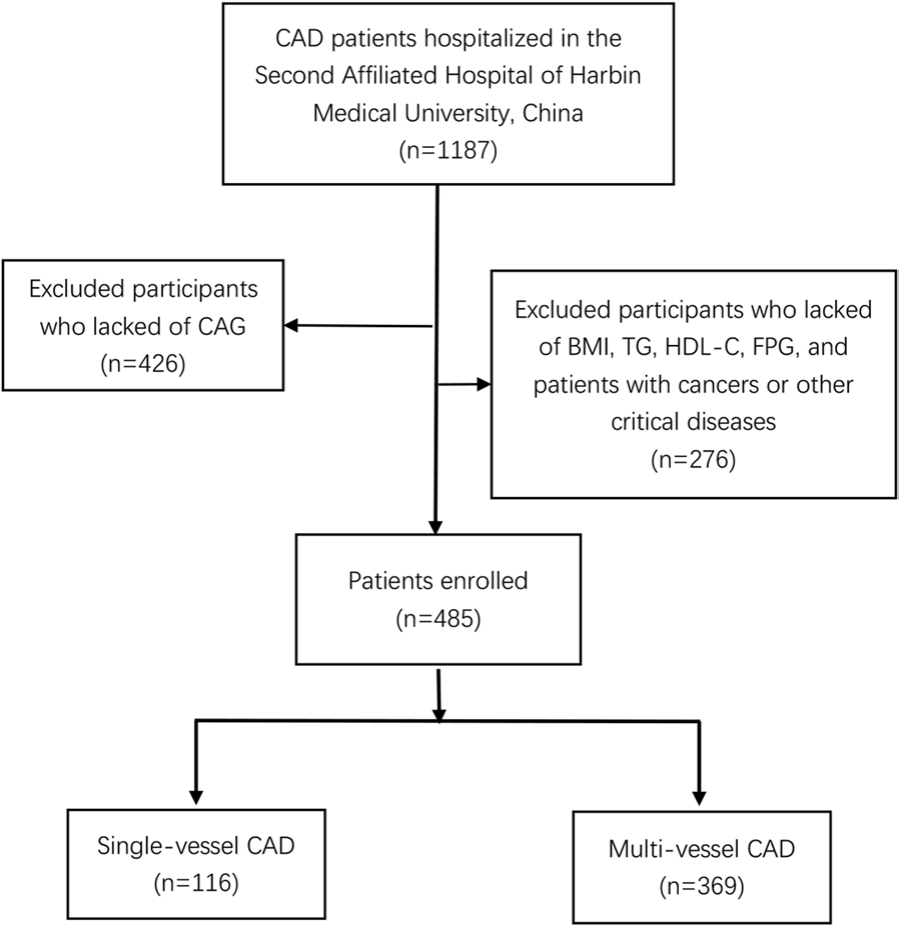
Flow chart of patient recruitment. CAD, coronary artery disease; CAG, coronary angiography; BMI, body mass index; FPG, fasting plasma glucose; TG, triglycerides; HDL-C, high-density lipoprotein cholesterol

This was a retrospective analysis, comprising 1187 CAD patients who visited the Second Affiliated Hospital of Harbin Medical University, China between August 2020 and August 2021. Patients were excluded from the study if they; had incomplete clinical data; were pregnant or lactating mothers; had serious infections, vital organ dysfunction or mental disease; and were younger than 18 years old. Finally, 485 subjects were included, according to the inclusion process illustrated in Fig. 1. The study was conducted in accordance with the Declaration of Helsinki, and was approved by the Ethics Review Committee of the Second Affiliated Hospital of Harbin Medical University. Informed consent was obtained from all subjects prior to inclusion in the study prior to inclusion in the study.

### Data collection

Clinical data collected from each subject included their age, sex, height, weight, history of diabetes mellitus (DM) and hypertension, as well as smoking and drinking status. A medical professional also collected blood samples from each subject’s fasting venous blood, which was subsequently used to determine various parameters including fasting plasma glucose (FPG), glycated hemoglobin A1c (HbA1c), total cholesterol (TC), triglycerides (TG), high-density lipoprotein cholesterol (HDL-C), low-density lipoprotein cholesterol (LDL-C), uric acid (UA), B-type natriuretic peptide (BNP), C-reactive protein (CRP), and creatinine (Cr). Echocardiographic data, including left atrial diameter (LAD), left ventricular end diastolic diameter (LVDd), left ventricular systolic diameter (LVDs), interventricular septal thickness (IVS), left posterior wall thickness (LVPW), and left ventricular ejection fraction (LVEF) were collected.

The severity of CAD can be assessed by the patient's clinical symptoms and auxiliary examinations. CAG is the ‘gold standard’ for the diagnosis of CAD, and more than 50% stenosis is directly related to the occurrence of CAD. So CAD was defined in our study as luminal stenosis of ≥ 50% in at least one major coronary artery (left anterior descending, left circumflex, and right coronary arteries), and the number of diseased vessels with ≥ 50% stenosis indicated the severity of CAD in patients. Patients in which only one major coronary artery was involved were considered to have single-vessel CAD, whereas patients with involvement of two or more major coronary arteries were considered to have multivessel CAD. Those without vascular lesions, but with left main coronary artery stenosis ≥ 50% were considered as two lesions [13].

### Definition of terms

Body mass index (BMI) was calculated as weight (kg) divided by the square of height (m^2^).

- TyG = Ln [TG (mg/dL) × FPG (mg/dL) ÷ 2] [8]
- TyG-BMI = TyG × BMI (kg/m^2^) [9]
- TG/HDL-C = TG (mg/dl) ÷ HDL-C (mg/dl) [18]
- METS-IR = Ln [(2 × FPG (mg/dl)) + TG (mg/dl)] × BMI (kg/m^2^) ÷ Ln [HDL-C (mg/dl)] [12].

### Participants stratification

Study subjects were grouped based on tertiles as follows;

- T1 group, TyG index < 8.777 (n = 161); T2 group, TyG index ≥ 8.777 to < 9.273 (n = 163), and T3 group, TyG index ≥ 9.273 (n = 161).
- B1 group, TyG-BMI index < 216.014 (n = 161); B2 group, TyG-BMI index ≥ 216.014 to < 247.645 (n = 163), and B3 group, TyG-BMI index ≥ 247.645 (n = 161).
- G1 group, TG/HDL-C ratio < 2.060; (n = 161); G2 group, TG/HDL-C ratio ≥ 2.060; to < 3.589 (n = 163), and G3 group, TG/HDL-C ratio ≥ 3.589 (n = 161).
- M1 group, METS-IR < 38.405 (n = 161); M2 group, METS-IR ≥ 38.405 to < 44.658 (n = 163), and M3 group, METS-IR ≥ 44.658 (n = 161).

### Statistical analysis

Statistical analysis of all data was performed using SPSS 26.0 (IBM Corp, New York, NY, USA) at significance level of *P* ≤ 0.05. Continuous variables that conformed to normal distribution were expressed as means ± standard deviation (x ± s), and compared between groups using the *t*-test or analysis of variance. Continuous variables with non-normal distribution were expressed as medians (P25–P75) and compared between groups using either the Mann–Whitney U or Kruskal–Wallis H tests. Categorical variables were presented as numbers (%), and compared between groups using the chi-squared or Fisher’s exact test. Odds ratios (ORs) and 95% confidence intervals (CIs) were calculated using logistic regression analysis. A receiver operating characteristic (ROC) curve was used for diagnostic value analysis.

## Results

### Baseline characteristics

Baseline characteristics of all subjects, including 116 and 369 patients with single- and multi-vessel CAD, are shown in Table 1. Summarily, patients with multi-vessel CAD were significantly older and had higher prevalence of diabetes and hypertension than their single-vessel counterparts (all *P* < 0.05). Moreover, subjects in the multi-vessel CAD group exhibited significantly higher HbA1c, FPG, TG, LAD, IVS, LVPW, BNP, TyG index, TyG-BMI index, TG/HDL-C ratio and METS-IR than their counterparts in the single-vessel group (all *P* < 0.05). However, patients in the multi-vessel CAD group had significantly lower HDL-C than those in the single-vessel CAD group (*P* < 0.05) (Table 1).

**Table 1:** Baseline characteristics of the study population

	Total (n = 485)	Single-vessel CAD (n = 116)	Multi-vessel CAD (n = 369)	*P*-value
Age (years)	60 (52, 67)	57 (52, 65)	61 (52.50, 67)	0.028
Male (n, %)	359 (74%)	86 (74.10%)	273 (74%)	0.974
BMI (kg/m^2^)	25.69 (23.53, 27.98)	25.24 (22.52, 26.82)	25.95 (23.88. 28.33)	< 0.001
DM (n, %)	185 (38.10%)	21 (18.10%)	164 (44.40%)	< 0.001
Smoking (n, %)	200 (41.20%)	56 (48.30%)	144 (39%)	0.077
Drinking (n, %)	67 (13.80%)	18 (15.50%)	49 (13.30%)	0.542
Hypertension (n, %)	225 (46.40%)	41 (35.30%)	184 (49.90%)	0.006
HbA1c (%)	6.10 (5.70, 7)	5.80 (5.50, 6.30)	6.20 (5.70, 7.40)	< 0.001
FPG (mmol/l)	7.47 (6.21, 10.14)	6.89 (5.99, 8.10)	7.68 (6.29, 10.68)	< 0.001
TC (mmol/l)	4.76 (3.98, 5.60)	4.76 (4.30, 5.49)	4.77 (3.94, 5.65)	0.684
TG (mmol/l)	1.31 (0.94, 1.81)	1.15 (0.82, 1.53)	1.38 (0.97, 1.89)	< 0.001
HDL-C (mmol/l)	1.12 (0.93, 1.29)	1.23 (1.06, 1.36)	1.08 (0.91, 1.26)	< 0.001
LDL-C (mmol/l)	3.20 (2.48, 3.86)	3.24 (2.66, 3.77)	3.18 (2.41, 3.91)	0.636
UA (umol/l)	368.90 (306.15, 448.95)	353.60 (301.45, 419.53)	376.70 (307.25, 458)	0.053
LAD (mm)	35.60 (32.50, 38.30)	34.20 (31.60, 36.50)	35.80 (32.90, 38.70)	0.001
LVDd (mm)	46.60 (43.60, 49.70)	46.60 (43.30, 48.80)	46.60 (43.80, 50.13)	0.156
LVDs (mm)	29.60 (25.60, 33.55)	29.40 (25, 32.80)	29.60 (25.90, 33.90)	0.271
IVS (mm)	10.80 (10.10, 11.60)	10.60 (9.90, 11.30)	10.90 (10.10, 11.80)	0.010
LVPW (mm)	10.50 (10, 11.20)	10.30 (10, 10.80)	10.50 (10, 11.23)	0.020
LVEF (%)	59 (52.45, 62)	58.80 (54.10, 62)	59.10 (52, 62)	0.706
BNP (pg/ml)	551 (165.50, 1514)	327.50 (137.50, 928)	702 (190.50, 1726.50)	0.001
D-dimer (ug/ml)	94 (52.50, 175)	86.50 (48, 188.25)	96 (56.50, 172.50)	0.319
CRP (mg/l)	6.14 (2.02, 13.20)	5.53 (1.63, 12.94)	6.36 (2.25, 13.23)	0.368
Cr (umol/l)	77 (65, 92)	75 (64.25, 85.75)	78 (65.50, 95)	0.057
TyG index	9.01 (8.65, 9.41)	8.77 (8.42, 9.06)	9.09 (8.71, 9.52)	< 0.001
TyG-BMI index	231.59 (210.41, 256.57)	213.89 (195.09, 237.29)	236.59 (213.63, 261.54)	< 0.001
TG/HDL-C ratio	2.65 (1.83, 3.99)	2.14 (1.53, 2.95)	3.04 (1.89, 4.27)	< 0.001
METS-IR	41.38 (37.20, 46.38)	38.22 (34.20, 41.91)	42.91 (37.93, 47.55)	< 0.001

Data are presented as median (interquartile range) or number (proportion, %)

CAD, Coronary artery disease; BMI, body mass index; DM, diabetes mellitus; FPG, fasting plasma glucose; HbA1c, glycated hemoglobin A1c; TC, total cholesterol; TG, triglycerides; HDL-C, high-density lipoprotein cholesterol; LDL-C, low-density lipoprotein cholesterol; UA, uric acid; LAD, left atrial diameter; LVDd, left ventricular end diastolic diameter; LVDs, left ventricular systolic diameter; IVS, interventricular septal thickness; LVPW, left posterior wall thickness; LVEF, left ventricular ejection fraction; BNP, B-type natriuretic peptide; CRP, C-reactive protein; Cr, creatinine; TyG index, triglyceride and glucose index; TyG-BMI index, triglyceride glucose-body mass index; TG/HDL-C ratio, triglyceride to high-density lipoprotein cholesterol ratio; METS-IR, metabolic score for insulin resistance

### Correlations among TyG index, TyG-BMI index, TG/HDL-C ratio, METS-IR and CAD severity

Taking the multi-vessel CAD as the dependent variable in the univariate logistic regression analysis, we found that DM, hypertension, BMI, HbA1c, FPG, TG, HDL-C UA, LAD, IVS, LVWP, TyG index, TyG-BMI index, TG/HDL-C ratio and MEST-IR were significantly associated with the risk of CAD (*P* < 0.05) (Table 2).

**Table 2:** Associations between CAD severity and risk factors

Variables	Multi-vessel coronary artery disease		
	OR (95% CI)	Β	*P*-value
Age	1.019 (0.999–1.039)	0.19	0.056
Sex			
Male	Reference		
Female	1.008 (0.626–1.623)	0.008	0.974
DM			
No	Reference		
Yes	3.619 (2.162–6.059)	1.286	< 0.001
Smoking			
No	Reference		
Yes	0.686 (0.451–1.044)	− 0.399	0.078
Drinking			
No	Reference		
Yes	0.834 (0.464–1.497)	− 0.182	0.543
Hypertension			
No	Reference		
Yes	1.819 (1.181–2.802)	0.598	0.007
BMI	1.217 (1.128–1.313)	0.197	< 0.001
HbA1c	1.568 (1.260–1.951)	0.450	< 0.001
FPG	1.183 (1.089–1.286)	0.168	< 0.001
TC	0.974 (0.820–1.157)	− 0.026	0.764
TG	1.872 (1.320–2.655)	0.627	< 0.001
HDL-C	0.153 (0.068–0.347)	− 1.876	< 0.001
LDL-C	0.960 (0.786–1.172)	− 0.041	0.689
UA	1.002 (1.000–1.004)	0.002	0.035
BNP	1.000 (0.999–1.001)	< 0.001	0.089
D-dimer	1.000 (0.999–1.001)	< 0.001	0.771
CRP	1.015 (0.977–1.055)	0.015	0.434
Cr	1.006 (0.999–1.013)	0.006	0.083
LAD	1.085 (1.031–1.143)	0.082	0.002
LVDd	1.044 (0.998–1.091)	0.043	0.058
LVDs	1.029 (0.994–1.065)	0.028	0.111
IVS	1.254 (1.059–1.486)	0.227	0.009
LVPW	1.253 (1.038–1.512)	0.225	0.019
LVEF	0.982 (0.957–1.009)	− 0.018	0.194
TyG index	3.170 (2.092–4.803)	1.154	< 0.001
TyG-BMI index	1.028 (1.019–1.036)	0.027	< 0.001
TG/HDL-C ratio	1.346 (1.165–1.555)	0.297	< 0.001
MEST-IR	1.166 (1.118–1.217)	0.154	< 0.001

OR, odds ratios; CI, confidence interval; β, regression coefficient; DM, diabetes mellitus; FPG, fasting plasma glucose; HbA1c, glycated hemoglobin A1c; BMI, body mass index; TC, total cholesterol; TG, triglycerides; HDL-C, high-density lipoprotein cholesterol; LDL-C, low-density lipoprotein cholesterol; UA, uric acid; LAD, left atrial diameter; LVDd, left ventricular end diastolic diameter; LVDs, left ventricular systolic diameter; IVS, interventricular septal thickness; LVPW, left posterior wall thickness; LVEF, left ventricular ejection fraction; BNP, B-type natriuretic peptide; CRP, C-reactive protein; Cr, creatinine; TyG index, triglyceride and glucose index; TyG-BMI index, triglyceride glucose-body mass index; TG/HDL-C ratio, triglyceride to high-density lipoprotein cholesterol ratio; METS-IR, metabolic score for insulin resistance

### Relationship between TyG index and CAD severity

Multi-vessel CAD was used as the dependent variable for logistic regression analysis, and there was no multicollinearity among the independent variables. We observed the correlation between IR-related indicators and multi-vessel CAD from the perspectives of continuous and classified variables by adjusting for different risk factors. One was not to adjust any risk factors; the second was to adjust the most common risk factors, age and gender; and the last was to adjust the risk factors screened by the univariate logistic regression. The results showed that TyG index was significantly correlated with multi-vessel CAD (*P* < 0.05). Then we grouped TyG index based on tertiles, the risk of multi-vessel CAD was higher in the T2 and T3 groups than in the T1 group, as shown in model 1 and model 2 (*P* < 0.05). The risk of multi-vessel CAD for patients in T3 was 2.985 times greater (95% CI 1.348–6.609; *P* = 0.007) than in patients with T1 group after adjusting for age, sex, DM, hypertension, HbA1c, BMI, HDL-C, UA, LAD, IVS, LVPW in model 3 (Table 3).

**Table 3:** Association between TyG index and CAD severity

Variables	Multi-vessel coronary artery disease					
	OR (95% CI)^a^	*P*-value	OR (95% CI)^b^	*P*-value	OR (95% CI)^c^	*P*-value
TyG index	3.170 (2.092–4.803)	< 0.001	3.378 (2.212–5.160)	< 0.001	2.280 (1.300–3.997)	0.004
T1	Reference		Reference		Reference	
T2	1.676 (1.039–2.703)	0.034	1.747 (1.075–2.840)	0.024	1.404 (0.812–2.428)	0.224
T3	4.770 (2.626–8.663)	< 0.001	5.322 (2.891–9.795)	< 0.001	2.985 (1.348–6.609)	0.007

OR, odds ratios; CI, confidence interval; TyG index, triglyceride and glucose index; T1: TyG index < 8.777; T2: 8.777 ≤ TyG index < 9.273; T3: 9.273 ≤ TyG index

^a^Model 1: Unadjusted

^b^Model 2: Adjusted for age and sex

^c^Model 3: Adjusted for age, sex, DM, hypertension, HbA1c, BMI, HDL-C, UA, LAD, IVS, LVPW

### Association between TyG-BMI index and CAD severity

Next, we found that the TyG-BMI index was significantly associated with increased risk of multi-vessel CAD (*P* < 0.05) (Table 4). Then, the TyG-BMI index was divided into three groups according to tertile, with the B1 group as a reference, B2 and B3 groups had a higher risk of multi-vessel CAD in model 4 and model 5 (*P* < 0.05). Compared with the B1 group in model 6 that adjusted for age, sex, DM, hypertension, HbA1c, HDL-C, UA, LAD, IVS, LVPW, B3 showed a 4.588 increased risk of multi-vessel CAD (95% CI 2.221–9.477; *P* < 0.001) (Table 4).

**Table 4:** Association between TyG-BMI index and CAD severity

Variables	Multi-vessel coronary artery disease					
	OR (95% CI)^a^	*P*-value	OR (95% CI)^b^	*P*-value	OR (95% CI)^c^	*P*-value
TyG-BMI index	1.028 (1.019–1.036)	< 0.001	1.030 (1.022–1.039)	< 0.001	1.024 (1.015–1.034)	< 0.001
B1	Reference		Reference		Reference	
B2	1.876 (1.163–3.026)	0.010	1.960 (1.205–3.187)	0.007	1.617 (0.961–2.723)	0.070
B3	5.937 (3.196–11.032)	< 0.001	7.577 (3.950–14.536)	< 0.001	4.588 (2.221–9.477)	< 0.001

OR, odds ratios; CI, confidence interval; TyG-BMI index, triglyceride glucose-body mass index; B1: TyG-BMI index < 216.014; B2: 216.014 ≤ TyG-BMI index < 247.645; B3: 247.645 ≤ TyG-BMI index

^a^Model 4: Unadjusted

^b^Model 5: Adjusted for age and sex

^c^Model 6: Adjusted for age, sex, DM, hypertension, HbA1c, HDL-C, UA, LAD, IVS, LVPW

### Association between TG/HDL-C ratio and CAD severity

The TG/HDL-C ratio was an independent risk factor for multi-vessel CAD (*P* < 0.05) (Table 5). When the TG/HDL-C was divided into three groups, in models 7 and 8, the risk of multi-vessel CAD in group G3 was 3.668-fold higher (95% CI 2.052–6.556; *P* < 0.001) and 4.075-fold higher (95% CI 2.255–7.363; *P* < 0.001) than in G1 group, respectively. Patients in group G3 had a 3.953-fold higher risk of multi-vessel CAD (95% CI 2.025–7.717; *P* < 0.001) in model 9 compared with those in group G1 after adjusting for age, sex, DM, hypertension, HbA1c, FPG, BMI, UA, LAD, IVS and LVPW (Table 5).

**Table 5:** Association between TG/HDL-C ratio and CAD severity

Variables	Multi-vessel coronary artery disease					
	OR (95% CI)^a^	*P*-value	OR (95% CI)^b^	*P*-value	OR (95% CI)^c^	*P*-value
TG/HDL-C ratio	1.346 (1.165–1.555)	< 0.001	1.378 (1.190–1.596)	< 0.001	1.355 (1.153–1.593)	< 0.001
G1	Reference		Reference		Reference	
G2	1.327 (0.824–2.139)	0.245	1.353 (0.836–2.191)	0.218	1.289 (0.767–2.167)	0.338
G3	3.668 (2.052–6.556)	< 0.001	4.075 (2.255–7.363)	< 0.001	3.953 (2.025–7.717)	< 0.001

OR, odds ratios; CI, confidence interval; TG/HDL-C ratio, triglyceride to high-density lipoprotein cholesterol ratio; G1: TG/HDL-C radio < 2.060; G2: 2.060 ≤ TG/HDL-C radio < 3.589; G3: 3.589 ≤ TG/HDL-C radio

^a^Model 7: Unadjusted

^b^Model 8: Adjusted for age and sex

^c^Model 9 adjusted for age, sex, DM, hypertension, HbA1c, FPG, BMI, UA, LAD, IVS, LVPW

### Association between METS-IR and CAD severity

Data shown in Table 6 indicated that the METS-IR was strongly associated with the risk of multi-vessel CAD (*P* < 0.05). Analysis of the METS-IR in three groups revealed that the risk of multi-vessel CAD for the model 10, model 11 and model 12 of M3 group were 12.725-fold (95% CI 5.591–28.965; *P* < 0.001), 16.320-fold (95% CI 6.983–38.142; *P* < 0.001), and 11.314-fold (95% CI 4.521–28.314; *P* < 0.001) higher than that of the M1 group (Table 6).

**Table 6:** Association between METS-IR and CAD severity

Variables	Multi-vessel coronary artery disease					
	OR (95% CI)^a^	*P*-value	OR (95% CI)^b^	*P*-value	OR (95% CI)^c^	*P*-value
METS-IR	1.166 (1.118–1.217)	< 0.001	1.180 (1.129–1.233)	< 0.001	1.160 (1.104–1.219)	< 0.001
M1	Reference		Reference		Reference	
M2	1.307 (0.823–2.075)	0.256	1.312 (0.818–2.104)	0.259	1.151 (0.694–1.911)	0.586
M3	12.725 (5.591–28.965)	< 0.001	16.320 (6.983–38.142)	< 0.001	11.314 (4.521–28.314)	< 0.001

OR, odds ratios; CI, confidence interval; METS-IR, metabolic score for insulin resistance; M1: METS-IR < 38.405; M2: 38.405 ≤ METS-IR < 44.658; M3: 44.658 ≤ METS-IR

^a^Model 10: Unadjusted

^b^Model 11: Adjusted for age and sex

^c^Model 12: Adjusted for age, sex, DM, hypertension, HbA1c, UA, LAD, IVS, LVPW

### The performance of TyG index, TyG-BMI index, TG/HDL-C ratio, and METS-IR in predicting the risk of CAD

**Fig. 2:**
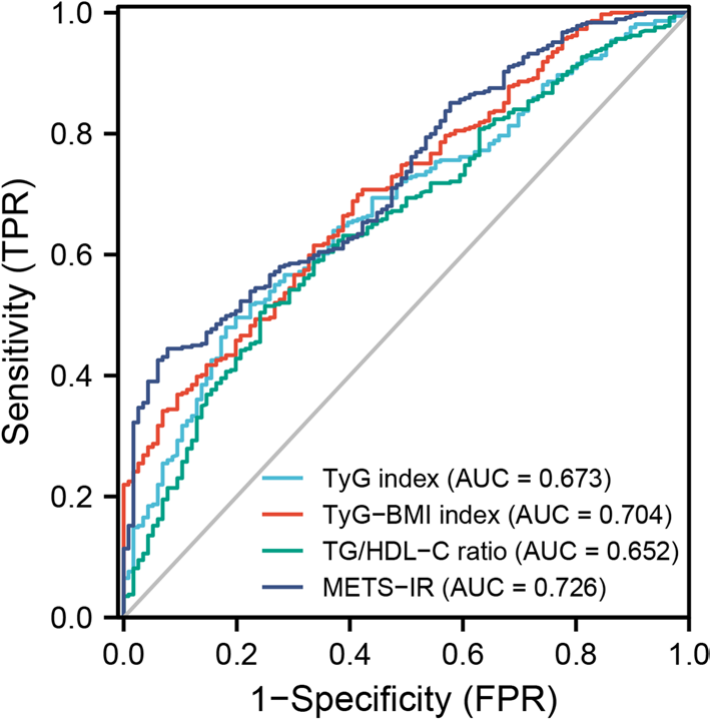
ROC curve for the use of TyG index, TyG-BMI index, TG/HDL-C ratio, METS-IR in the detection of multi-vessel CAD. TyG index, triglyceride and glucose index; TyG-BMI index, triglyceride glucose-body mass index; TG/HDL-C ratio, triglyceride to high-density lipoprotein cholesterol ratio; METS-IR, metabolic score for insulin resistance

The receiver operating characteristic (ROC) curves for multi-vessel CAD and TyG index, TyG-BMI index, TG/HDL-C ratio, METS-IR are shown in Fig. 2. The area under the curve (AUC) of the ROC plots for the TyG index was 0.673 (95% CI 0.620–0.726; *P* < 0.001), 0.704 (95% CI 0.652–0.755; *P* < 0.001) for the TyG-BMI index, while those for TG/HDL-C ratio and METS-IR were 0.652 (95% CI 0.597–0.708; *P* < 0.001) and 0.726 (95% CI 0.677–0.775; *P* < 0.001), respectively (Table 7).

**Table 7:** Comparison of the predictive value of the TyG index, TyG-BMI index, TG/HDL-C ratio, and METS-IR for the presence of CAD

Variable	AUC	95% CI	Cut-off value	Sensitivity (%)	Specificity (%)
TyG index	0.673	0.620–0.726	10.422	48.0	81.9
TyG-BMI index	0.704	0.652–0.755	210.120	70.0	58.6
TG/HDL-C ratio	0.652	0.597–0.708	1.665	52.0	75.0
METS-IR	0.726	0.677–0.775	44.350	44.4	92.2

AUC, area under the curve; CI, confidence interval; TyG index, triglyceride and glucose index; TyG-BMI index, triglyceride glucose-body mass index; TG/HDL-C ratio, triglyceride to high-density lipoprotein cholesterol ratio; METS-IR, metabolic score for insulin resistance

## Discussion

In the present study, we provide the first report of the relationship between TyG-BMI index and CAD severity in patients. Notably, this is also the first study comparing the value of TyG index, TyG-BMI index, TG/HDL-C ratio, and METS-IR in predicting CAD severity.

Previous studies have associated the increase of obesity to the rise in the incidence and prevalence of IR as well as related CVD, and there is a mutually reinforcing effect [19]. IR has been shown to be an important risk factor for CAD, with its degree positively associated with CAD severity [20, 21]. Furthermore, studies have demonstrated that patients with multi-vessel CAD have worse disease than their single-vessel CAD counterparts [22]. In the present study, we evaluated whether IR related indicators could predict CAD severity, with the aim of generating novel insights to guide early prevention of the disease, as well as reduce risk and improve patient prognosis. We employed the hyperinsulinemic–euglycemic clamp technique to evaluate IR [23], although its clinical use is limited due to experimental complexity and high cost [24]. The evaluation indicators that are simpler, cheaper and can be widely carried out need to be found. Previous studies have demonstrated that TyG index, TyG-BMI index, TG/HDL-C ratio, and METS-IR are not only effective indicators for evaluating IR [8–12], but may also be correlated with CAD severity [25–28].

Results of the present study showed that patients in the multi-vessel CAD group had significantly higher TyG index, TyG-BMI index, TG/HDL-C ratio and METS-IR than those in the control group. Notably, these four indicators were still independent risk factors for multi-vessel CAD even after adjusting for confounding factors. Next, we divided the patients into three groups, based on tertiles, and found that those with the highest tertile of TyG index, TyG-BMI index, TG/HDL-C ratio and METS-IR were significantly associated with the risk of multi-vessel CAD compared to those with the lowest tertile. Collectively, these results indicated that the increase of these four indicators was related to the significantly increased risk of multi vessel CAD.

TyG-BMI index is a simple, powerful and clinically useful alternative marker for early detection of IR [9]. Previous studies have associated TyG-BMI index with development of coronary atherosclerosis [26] as well as increased risk of cardiovascular ischemic stroke [29]. To date, however, nothing is known regarding the relationship between TyG-BMI index and CAD severity. In the present study, we focused on the relationship between TyG-BMI index and CAD severity, and found that TyG-BMI index was significantly associated with a high risk of multi-vessel CAD, with a good predictive value. Notably, an increase in the index resulted in a higher risk of multi-vessel CAD. Therefore, TyG-BMI index is expected to become a predictor of CAD severity and a key target of future clinical applications.

Several studies have shown that TyG index is also a simple method for detecting IR, thus a crucial factor for early identification of the high risk of cardiovascular events. Moreover, it is a robust marker for diagnosis of metabolic syndrome [30–32]. Results from a large-scale retrospective analysis conducted in South Korea revealed that the group with the highest TyG index had a higher risk of stroke and myocardial infarction [31]. On the other hand, Su et al. [13] demonstrated that TyG index was associated with CAD severity, while Wang et al. [2] showed that TyG index, as an indicator for evaluating IR, may be a valuable predictor of CAD severity. Our results were consistent with findings from the above-mentioned studies. Notably, it was evident that the TyG index was associated with CAD severity, with the highest tertile associated with a significantly higher risk compared to the lowest one.

High TG and low HDL-C, specific cardiometabolic features of atherosclerotic dyslipidemia, have been associated with both development of metabolic syndrome and the risk of CHD [33–35]. Several studies have shown that high TG/HDL-C significantly increases the risk of CVD [36, 37]. Wu et al. [14] also confirmed that TG/HDL-C ratio was an independent predictor for the existence of CAD, although this parameter exhibited no statistical significance in predicting the CAD severity after adjusting for confounding factors. This was in contrast to results from the present study, in which TG/HDL-C ratio was still an independent predictor of multi vessel CAD even after adjusting for confounding factors. The discrepancy in results between these studies may be due to differences in study regions.

In 2018, Bello-Chavolla et al. [12] proposed METS-IR as a promising new indicator for assessing cardiometabolic risk and screening insulin sensitivity. Results of a Korean population without DM revealed that higher METS-IR had better predictive value for ischemic heart disease than metabolic syndrome [28]. Results from a prospective cohort study also showed the elevated METS-IR was independently associated with CVD events [38]. To date, only one study has correlated METS-IR and CAD severity. Notably, this study showed that METS-IR could predict the severity of CAD, and had the highest predictive value compared with TyG index and TG/HDL-C ratio [14]. This is consistent with the results of the present study, which showed that METS-IR was not only an independent risk factor for multi-vessel CAD but also had the highest predictive value, affirming the association between METS-IR and CAD severity.

Previous studies have shown that drug interventions, such as hypoglycemic agents, antiplatelet drugs, lipid-lowering drugs and antihypertensive drugs, may affect the results [39, 40]. However, some studies also revealed that TyG index and METS-IR were still predictors of CAD after adjusting for the effect of drugs [2, 13, 14]. Medication history was not included in our study due to lack of detailed data. However, we excluded some factors that are closely related to drug use, such as blood lipids, blood glucose and blood pressure. This might weaken the effect of the drug on the results. In the future, we will expand the database and further observe the relationship between these indicators and CAD.

In the present study, we analyzed alternative IR indicators and found that all four indicators were significantly associated with CAD severity. ROC curves showed that METS-IR had the highest efficiency in predicting CAD severity, followed by TyG-BMI index. Moreover, TyG-BMI index had highest sensitivity and METS-IR had highest specificity, co-prediction of TyG-BMI index and METS-IR can make up for their respective defects and obtain more accurate results, which provided ideas for clinical judgment of patients' condition in the future.

## Strengths and limitations

In terms of strength of our study is the first investigation to show a relationship between TyG-BMI index and CAD severity. In addition, this is the first study to compare the value of TyG index, TyG-BMI index, TG/HDL-C ratio, and METS-IR in predicting CAD severity. However, this study still had some limitations. Firstly, this was a single-center study, which might have potential bias. Secondly, we could not determine the existence of the causality due to the inherent limitations associated with studies with a retrospective design. Thirdly, the study had a relatively small sample size, which might have some influence on the results. And the application of TyG-BMI index was limited to CAD population. Therefore, we need to expand the scope of the study and conduct a larger sample size, multi-center and prospective research to verify our findings.

## Conclusion

In summary, TyG-BMI index was significantly associated with both severity of CAD and occurrence of multi-vessel CAD, as evidenced by a strong relationship between the TyG index, TG/HDL-C ratio and METS-IR with CAD severity. Comparison of the four non-insulin-based IR indexes showed that the METS-IR had the highest predictive value, followed by TyG-BMI index. By conducting research in different groups from different angles, we can have a deeper understanding of these indicators, select the more valuable one for clinical application. These indicators are expected to be effective, simple and inexpensive predictors of CAD severity in clinical practice. Monitoring these indicators may help to assess the patient's condition in advance, and make more appropriate risk management and healthcare decisions.

## Data Availability

The datasets used and/or analyzed during the current study are available from the corresponding author on reasonable request.

## Abbreviations

CAD: Coronary artery disease
CHD: Coronary heart disease
IR: Insulin resistance
CVD: Cardiovascular disease
CAG: Coronary angiography
BMI: Body mass index
DM: Diabetes mellitus
FPG: Fasting plasma glucose
HbA1c: Glycated hemoglobin A1c
TC: Total cholesterol
TG: Triglycerides
HDL-C: High-density lipoprotein cholesterol
LDL-C: Low-density lipoprotein cholesterol
UA: Uric acid
LAD: Left atrial diameter
LVDd: Left ventricular end diastolic diameter
LVDs: Left ventricular systolic diameter
IVS: Interventricular septal thickness
LVPW: Left posterior wall thickness
LVEF: Left ventricular ejection fraction
BNP: B-type natriuretic peptide
CRP: C-reactive protein
Cr: Creatinine
TyG index: Triglyceride and glucose index
TyG-BMI index: Triglyceride glucose-body mass index
TG/HDL-C ratio: Triglyceride to high-density lipoprotein cholesterol ratio
METS-IR: Metabolic score for insulin resistance
OR: Odds ratios
CI: Confidence interval
β: Regression coefficient
ROC: Receiver operating characteristic
AUC: Area under the curve
